# Intergenomic Arms Races: Detection of a Nuclear Rescue Gene of Male-Killing in a Ladybird

**DOI:** 10.1371/journal.ppat.1000987

**Published:** 2010-07-08

**Authors:** Tamsin M. O. Majerus, Michael E. N. Majerus

**Affiliations:** 1 Institute of Genetics, Queens Medical Centre, University of Nottingham, Nottingham, United Kingdom; 2 Department of Genetics, University of Cambridge, Cambridge, United Kingdom; University of California, Riverside, United States of America

## Abstract

Many species of arthropod are infected by deleterious inherited micro-organisms. Typically these micro-organisms are inherited maternally. Consequently, some, particularly bacteria of the genus *Wolbachia*, employ a variety of strategies that favour female over male hosts. These strategies include feminisation, induction of parthenogenesis and male-killing. These strategies result in female biased sex ratios in host populations, which lead to selection for host factors that promote male production. In addition, the intra-genomic conflict produced by the difference in transmission of these cytoplasmic endosymbionts and nuclear factors will impose a pressure favouring nuclear factors that suppress the effects of the symbiont. During investigations of the diversity of male-killing bacteria in ladybirds (Coccinellidae), unexpected patterns of vertical transmission of a newly discovered male-killing taxon were observed in the ladybird *Cheilomenes sexmaculata*. Initial analysis suggested that the expression of the bacterial male-killing trait varies according to the male(s) a female has mated with. By swapping males between females, a male influence on the expression of the male-killing trait was confirmed. Experiments were then performed to determine the nature of the interaction. These studies showed that a single dominant allele, which rescues male progeny of infected females from the pathological effect of the male-killer, exists in this species. The gene shows typical Mendelian autosomal inheritance and is expressed irrespective of the parent from which it is inherited. Presence of the rescue gene in either parent does not significantly affect the inheritance of the symbiont. We conclude that *C. sexmaculata* is host to a male-killing γ-proteobacterium. Further, this beetle is polymorphic for a nuclear gene, the dominant allele of which rescues infected males from the pathogenic effects of the male-killing agent. These findings represent the first reported case of a nuclear suppressor of male-killing in a ladybird. They are considered in regard to sex ratio and intra-genomic conflict theories, and models of the evolutionary dynamics and distribution of inherited symbionts.

## Author Summary

Normally, in sexually reproducing organisms, the sex ratio (ratio of males to females) is 1∶1. However, examples are known where this is not the case and there are more females than males in a population. Extreme bias in sex ratio can lead to females failing to find a mate. We studied *Cheilomenes sexmaculata*, a ladybird species that has females that produce more female than male offspring. In aphid-eating ladybirds, this phenomenon has been widely reported and is known to be due to the presence of bacteria that live inside the mother and are passed via her eggs to her offspring. In eggs destined to become male, the bacteria kill the embryo by some unknown mechanism. This is known as male-killing. Female offspring develop normally. Evolutionary theory predicts that in such systems, the genome of the host can fight back if a variant arises that stops the bacteria killing male offspring. In *C. sexmaculata* we found females that carried the male-killer but the sex ratio of their offspring depended on the male that they mated with. We carried out breeding tests to show that some ladybirds had a version of a gene that rescued the male offspring from the pathological effects of the male-killer.

## Introduction

Cytoplasmic sex ratio distorters have been reported from many invertebrates [1]. One group of distorters comprises a diverse array of bacteria which distort the secondary sex ratio of their hosts towards females by killing male hosts early in embryogenesis [2], [3]. Infected females gain an advantage over uninfected females via inbreeding avoidance, resource reallocation or reduction in sibling competition. Theory predicts that, within populations biased towards females as a result of the action of maternally inherited cytoplasmic sex ratio distorters with incomplete vertical transmission, selection will favour autosomal sex ratio compensation [4], [5]. This could occur by distortion of the primary sex ratio or by distortion of the secondary sex ratio towards males, if loss of female offspring is compensated for by increased fitness of male progeny. No such case has been demonstrated in a diploid harbouring a male-killer (but see data of male-biased families in [6], [7]. In addition, selection may favour the evolution of autosomal genes that reduce the vertical transmission or the phenotypic effects of sex ratio distorting bacteria [8]. Autosomal genes that suppress the sex ratio phenotype are known for both cytoplasmic male sterility in plants [9] and sex chromosome meiotic drive in dipterans [10]. They are also suspected in the woodlouse *Porcellinoides pruinosus* which hosts a feminising *Wolbachia* [11]. A nuclear suppressor of male-killing could kill the male-killer, or reduce its vertical transmission, or prevent the symbiont from killing males, either by blocking male host recognition, or by blocking the killing act. Such a suppressor has been reported in *Drosophila prosaltans* where it is suggested there is a recessive allele that prevents transmission of the male-killer [12]. A suppressor conferring resistance has been demonstrated the butterfly *Hypolimnas bolina* infected with the male-killing *Wolbachia* strain wBol1, where infected Southeast Asian *H. bolina* produce a 1∶1 sex ratio [13]. It has been suggested that the widespread occurrence of males testing positive for known male-killers found via PCR screening of samples of 21 ladybird species, could be indicative of nuclear suppression [14]. Suppressors at fixation might also explain the findings in *Drosophila recens* and *Ephestia cautella*, where the *Wolbachia* strains they harbour cause male-killing when transferred to con-generic host species, although not in the original species [15], [16].

*Cheilomenes sexmaculata* harbours a male-killer [17]. This male-killer is transovarially transmitted, is horizontally transferable in haemolymph by microinjection and curable by both high temperature and tetracycline treatment [17], [18]. The causative agent associated with male-killing has previously been reported to be similar to that causing male-killing in *Harmonia axyridis* [18], a *Spiroplasma* [19].

## Results

Phenotypic assessments, based on egg hatch rates and progeny sex ratio, of a small sample from Tokyo showed that two of 15 matrilines had traits consistent with male-killing, with less than 50% of eggs hatching and female-biased progeny sex ratios (family Mk1 - 7 males, 73 females; family Mk2 - 2 males, 14 females) prior to antibiotic (tetracycline) treatment. Antibiotic treatment effected a cure of the trait, egg hatch rates and the proportion of males both increasing after treatment (Mk1 - 71 males, 76 females; Mk2 - 87 males, 93 females).

**Figure 1:**
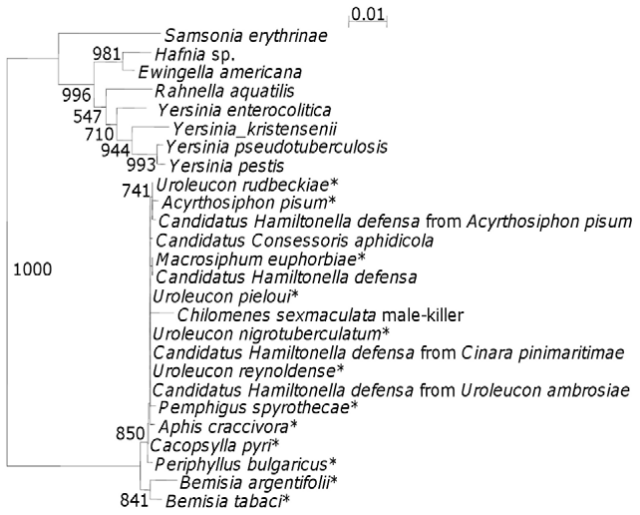
16S rDNA phylogenetic tree indicating the position of the *C. sexmaculata* male-killer amongst closely related bacteria. The numbers indicate bootstrap values (values within the clade containing the *C. sexmaculata* male-killer are low (most <500) and are not shown). * indicates secondary symbiont host name.

Identity of the male-killer was established by PCR amplification of the 16S rDNA gene using general eubacterial primers 27f and 1495r [20], cloning [21] and sequencing the gene. Briefly, genomic DNA was extracted [19] from females producing female biased sex ratios from both male-killer (m-k) lines (parental females, F_1_ and F_2_ progeny), from F_1_ eggs of both m-k lines, from F_1_ and F_2_ females from a normal (N) sex ratio line (N12) that never produced a biased sex ratio (five generations, 37 families), and from F_1_ and F_2_ progeny from antibiotic treated females from both m-k lines. The m-k line females from all three generations, the m-k eggs, but not the N line or progeny from antibiotic treated females, generated a 16S rDNA PCR product, which was then cloned and sequenced (1517 bp). A majority rule consensus sequence was generated for the bacterium to account for PCR errors (total 22 sequences, of a total of 26 sequences generated at this stage, 3 contaminant *Streptococcus lactis* and one pGEM non-transformant also sequenced). The sequence produced was submitted to EMBL (accession number AJ272038). This was compared with sequences in the EMBL, Genbank, DDBJ and PDB databases using BLASTN [22]. The sequence was identified as a γ-proteobacterium with closest similarity to a variety of secondary symbionts, of aphids (97-98% identity), in particular *Hamiltonella defensa* (98% identity), of whiteflies, particularly of *Bemisia tabaci* (97% identity) and to a number of bacteria of the genus *Yersinia*. To investigate these relationships further, a phylogeny was produced using 16S rDNA sequences from 25 of the closest matches from the BLASTN search (Figure 1). It is interesting to note that the male-killer clearly falls within the clade of aphid secondary symbionts and is distinct from both the whitefly symbiont clade and the *Yersinia* clade. The tree is not particularly informative regarding relationships within the aphid symbiont clade, with almost all of the bootstrap values being below 500. However, it is certainly suggestive of horizontal transfer of symbionts both between aphid species, as has been reported [23], [24], [25] and between prey and predator species as has been suggested for other coccinellid male-killers [26], [27].

The 16S rDNA phylogeny highlights one further interesting feature of this male-killer, as it supports the suggestion of horizontal transfer of symbionts with a change in phenotype between the alternative hosts. Secondary symbionts are known to confer protection to their hosts against natural enemies [25 for review], but natural enemy protection co-occurring with reproductive parasitism in a clade has to date only been established for *Wolbachia* [28].

This finding represents the second instance of a γ-proteobacterium causing male-killing [29], and the first recorded instance in a coccinellid. To confirm the absence of other known male-killers, in particular *Spiroplasma*, but also *Rickettsia*, *Wolbachia* and *Flavobacteria*, specific PCR assays were carried out on individuals from both male-killer lines, from the parental and F_1_ generations. In all cases these tests proved negative [27]. The male-killer in this sample of *C. sexmaculata* is thus not the same as that identified from *H. axyridis* [19], as has previously been assumed [18].

Crosses designed to test the inheritance of the trait, involving F_1_ females from the two putative male-killer lines (Mk1 and Mk2), with males from normal sex ratio matrilines produced unexpected progeny sex ratios (Table 1). All three crosses using males from one line (N5) produced significantly female-biased sex ratios, one (Mk1.1) being almost all female, the others (Mk1.4 and Mk2.3) giving approximately 2∶1 ratios of females to males. The remaining seven crosses, involving males from two other lines (N1 and N8) produced normal (approximately 1∶1) sex ratios.

**Table 1:** Outcome of crosses designed to test the inheritance of the m-k trait in two matrilines of *C. sexmaculata*.

Cross number	Origin of female and male parents (female first)	Egg hatch rate	Number of progeny	Proportion males
**Mk1.1**	**Mk1 x N5**	0.52	**75**	**0.013**
Mk1.2	Mk1 x N1	0.79	69	0.464
Mk1.3	Mk1 x N1	0.75	67	0.448
**Mk1.4**	**Mk1 x N5**	0.68	**85**	**0.341**
Mk1.5	Mk1 x N8	0.75	83	0.470
Mk2.1	Mk2 x N1	0.86	73	0.603
Mk2.2	Mk2 x N8	0.22	24	0.583
**Mk2.3**	**Mk2 x N5**	0.53	**56**	**0.339**
Mk2.4	Mk2 x N1	0.85	87	0.494
Mk2.5	Mk2 x N8	0.31	48	0.5

Progeny results (egg hatch rates and sex ratio) from initial matings of F_1_ females from two male-killer matrilines mated to males from normal sex ratio families.

Crosses given in bold show a significantly female-biased sex ratio, with egg hatch rates consistent with the death of a proportion of male embryos.

These data suggest either an exceptionally low and variable vertical transmission of the sex biasing trait, or paternal influence on progeny sex ratios.

**Figure 2:**
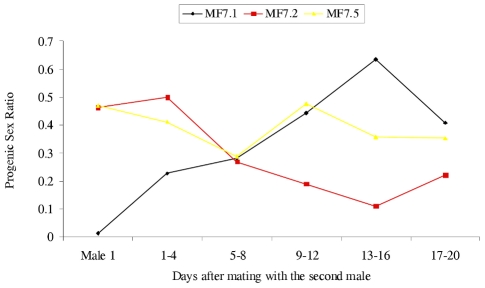
The effect of changing the male on progeny sex ratios produced by *C. sexmaculata* females. Progeny sex ratios of three F_1_ females, from a female biased matriline (Mk1) each mated twice. Male 1 indicates progeny from the first mating (see Table 1). Sex ratios after mating with the second male are given in four-day blocks. See Table S1 for data upon which the Figure is based.

To test the latter possibility, male parents were transferred between some of the crosses, the male (N5) from Mk1.1 being mated multiply to the female from Mk1.2 and once to the female from Mk1.5, and the male (N1) from Mk1.3 being mated to the Mk1.1 female. In all cases the progeny sex ratios changed following these additional matings (Figure 2), that of Mk1.1 rising from 0.013 to 0.355, with those of Mk1.2 and Mk1.5 becoming significantly female-biased. The Mk1.2 female, mated multiply to the second male produced a strong female bias from three days after introduction of the new male and for the remainder of her life. However, the Mk1.5 female, mated just once to a second male, again after a lag (four days) produced a strong and significant female bias (14 male∶42 female) over four days, before her progeny sex ratio reverted towards normality. This reversion suggests that sperm from the singly mated second male was used for a block of time before utilisation reverted to sperm from the multiply mated first male.

Extended replication of this mate swapping procedure using both male-killing matrilines showed that changes in the sex ratio following change of male were reversible if males were changed back (data available on request). These data suggest the presence of a factor, acting through sperm or some other element in the ejaculate, that inhibits the male-killing action of the bacterium. The initial data could be explained by a unifactorial dominant nuclear gene.

Four questions were addressed to test this hypothesis and detail the nature of the suppressor system.

- Is the suppressor inherited in a Mendelian fashion?
- Is expression of the suppressor affected by the sex of the parent from which it is inherited?
- Does the action of the suppressor affect the inheritance of the male-killer in female hosts?
- Do ‘rescued’ males carry the male-killer?

To address i) and ii) on the basis of the initial hypothesis, expected progeny sex ratios from monogamous crosses involving individuals of alternative genotypes with respect to both the male-killer and the suppressor locus (alleles: suppressor = *res^+^*, non suppressor = *res^−^*) were calculated (Table 2).

**Table 2:** Results of single pair crosses of individuals of inferred male-killer status and suppressor gene genotype.

Cross	Genotype of female parent	Genotype of male parent	Number of progeny	Observed sex ratio	Expected sex ratio	Ranges of Uncertainty
Mk1.7	mk, *res^−^res^−^*	*res^−^res^−^*	12	0	0.0–0.167	0–0.250
Mk1.8	mk, *res^−^res^−^*	*res^−^res^−^*	20	0	0.0–0.167	0–0.250
Mk1.11	mk, *res^−^res^−^*	*res^−^res^−^*	27	0.222	0.0–0.167	0–0.222
Mk1.1.1	mk, *res^−^res^−^*	*res^−^res^−^*	10	0.1	0.0–0.167	0–0.300
Mk1.1.2	mk, *res^−^res^−^*	*res^−^res^−^*	17	0	0.0–0.167	0–0.235
Mk1.1.3	mk, *res^−^res^−^*	*res^−^res^−^*	45	0.133	0.0–0.167	0–0.200
Mk1.1.4	mk, *res^−^res^−^*	*res^+^res^−^*	102	0.363	0.333–0.375	0.265–0.451
Mk1.1*.5	mk, *res^+^res^−^*	*res^−^res^−^*	99	0.364	0.333–0.375	0.263–0.455
Mk1.10	mk, *res^−^res^−^*	*res^+^res^−^*	216	0.329	0.333–0.375	0.287–0.421
Mk1.1*.6	mk, *res^+^res^−^*	*res^+^res^−^*	77	0.442	0.429–0.444	0.325–0.545
Mk1.1.4.9	mk, *res^+^res^−^*	*res^+^res^−^*	234	0.419	0.429–0.444	0.372–0.500
Mk2.3.3.1	mk, *res^+^res^+^*	*res^−^res^−^*	47	0.553	0.5	0.362–0.638
Mk2.3.1.2	mk, *res^−^res^−^*	*res^+^res^+^*	63	0.460	0.5	0.381–0.619

The expected sex ratio is based on the suppressor gene being a normal Mendelian locus, with the suppressor allele (*res^+^*) dominant to the non-suppressor allele (*res^−^*), and on a vertical transmission efficiency ranging from 0.8–1. The ranges of uncertainty indicate the 95% confidence limits of the expected sex ratios, incorporating binomial sampling and calculated by simulation. All sex ratios are given as proportion male. The generation of the maternal parent in a cross is indicated by number of full stops + 1. * indicates that the individual used was offspring of a second male mated to the Mk1.1 female. See Table S2 for further detail of the matrilines. All females were shown, by *post hoc* sequence analysis, to carry the male-killer.

Crosses involving individuals of inferred suppressor genotype, the females being F_1_, F_2_ or F_3_ from male-killer matrilines, produced progeny sex ratios consistent with expectation on the basis of Mendelian inheritance (Table 2). The results indicate that the expression of the suppressor is not affected by the sex of the parent from which the suppressor is inherited. For example, cross Mk1.1.4.9, in which both parents were heterozygous for the suppressor gave a progeny sex ratio of 0.419 (n = 234), close to expectation and significantly different from both the maximum expected sex ratio if only paternally derived suppressors are expressed (χ^2^_1_ = 4.869, p<.05), and from a 1∶1 sex ratio (χ^2^_1_ = 6.171, p<0.05).

Mk1 and Mk2 were maintained for five generations, 118 families (minimum number of progeny  = 10; mean number of progeny  = 62.7) being reared. In 51 of these the genotype with respect to the rescue gene locus was inferred for both parents prior to progeny being obtained. In 46 families, results were consistent with the theorised autosomal (or pseudoautosomal) nature of the locus. The locus is not sex-linked. In the remaining five, 16S rDNA sequence analysis, performed *post hoc*, showed that the female parent lacked the male-killer. This proportion of revertants is roughly consistent with the estimated vertical transmission efficiency, *a*, of the male-killer seen in Mk1 (*a* = 0.89) and Mk2 (*a* = 0.83).

To address question iii), two female F_2_ progeny from predominantly female families of both the Mk1 and Mk2 matrilines were crossed to homozygous *res^+^* males. The progeny sex ratio in each of these families was close to 1∶1. *res^+^res^−^* female offspring from these crosses were mated to *res^−^res^−^* males. Of eight crosses, one produced a normal sex ratio, the remainder producing ratios approximating to 1 male: 2 female (data not shown). Given the vertical transmission efficiency of this male-killer, these results show that the male-killer is inherited through females, even when its pathological effect on males has been suppressed. Verification was obtained by sequencing the 16S rDNA PCR product from parents and six progeny (3 male, 3 female) of two of the families that produced a 1 male: 2 female sex ratio. Female parents, all male progeny and five of the six female progeny were shown to bear the male-killer.

Verification that the suppressor acts as a rescue gene was obtained by demonstrating that adult males from suppressed male-killer females produced 16S rDNA PCR product with the same sequence as the γ-proteobacterium identified as the male-killer (question iv)). Two male progeny from two m-k families, one of which produced a 1∶2 sex ratio and one which produced a normal sex ratio (Mk1.4 and Mk2.5, respectively) were submitted to the same PCR cloning and sequencing protocol as used for the initial identification of the male-killer. In each case the γ-proteobacterium was shown to be present. The male-killer is thus present but not expressed in ‘rescued’ males.

Crosses of such males, inferred to be *res^+^res^−^* and to carry the male-killer, to *res^−^res^−^* females lacking the male-killer produced normal progeny sex ratios. The male-killer is thus not inherited from males. Taken together, these results suggest that the endosymbiont is not killed by the suppressor. Rather the suppressor acts as a rescue gene for males.

## Discussion

The discovery of a nuclear gene that rescues males from the pathological effects of a maternally inherited bacterium that otherwise kills males, to the benefit of the males' female siblings that carry and vertically transmit the bacterium, is in accord with theories of sex ratio [30], [31] and intra-genomic conflict [4], [5]. Selection favouring a suppressor gene will be a direct consequence of sex ratio distortion. Autosomal genes that act against sex ratio distorters have been recorded in isopods infected with feminising *Wolbachia* and in a butterfly infected with male-killing *Wolbachia*. In *Armadillium vulgare*, the main effect of such genes is to reduce bacterial transmission to progeny [32]. In contrast, in *P*. *pruinosus*, autosomal genes are conjectured to prevent the feminising effect of *Wolbachia* [11]. This is analogous to the situation in *C. sexmaculata* and *H*. *bolina* [13] where observations are compatible with a single, dominant autosomal locus suppressing the male-killing effect of the bacteria.

Most models considering the evolutionary interactions between sex ratio distorting symbionts and suppressors are based on the assumption that the suppressor will kill the symbiont or reduce its vertical transmission [33], [34]. The dynamics of a male rescue gene may be quite different, and will depend on its cost, if any, in the absence of the male-killer. A male-killer in the presence of a male rescue gene should be selected against, due to the cost on hosts of carrying the male-killer and the lack of fitness advantages to infected females resulting from male death [3]. Such a male-killer then has several alternative fates. It may be selected to extinction. It may become polymorphic, male killer prevalence being determined by its transmission dynamics and fitness compensation and the costs to hosts of both it and the rescue gene. It may circumvent the rescue gene by evolving a different mechanism to kill males (cf. the double feminising effect of *Wolbachia* in *A. vulgare* [35]). Finally, it may reduce its cost on hosts, becoming costless (persistence would require vertical transmission close to 1) or even beneficial - cytoplasmic male-killers are an exquisite testing ground for theories of virulence [3].

It is interesting to compare the situation in *C. sexmaculata* with that in *H. bolina*. In the latter, the suppressor has recently and rapidly spread to fixation in Southeast Asian populations, but is absent from Polynesian populations [13]. A recent model [36] examines the dynamics of such systems with reference to host suppressors of male-killing and *Wolbachia* that are also able to induce cytoplasmic incompatibility (CI). Here the model predicts that (in the absence of CI, and so pertinent to this study) the maximum cost of a dominant suppressor of male-killing that allows invasion of a host population will increase as the male-killer prevalence increases. The model also predicts (in the absence of CI) that a costly suppressor that does invade will become polymorphic and the frequency of the male-killer will be reduced. Further, the authors suggest that polymorphic suppressors of the male-killing action of non-*Wolbachia* male-killers (not known to induce CI) should be more common than of *Wolbachia* male-killers. Our findings fit well with this model.

In contrast to the male-killer in *C. sexmaculata*, the male-killing *Wolbachia* in *H. bolina* may also cause CI and where this occurs the model demonstrates that the dynamics of the system are altered, with corresponding changes in the rate of spread, fixation and frequency of infection that depend on the level of CI, the cost of the suppressor, the transmission efficiency and the initial male-killer frequency. Suppressor spread may be inhibited, where there is CI, but where a suppressor does spread it is predicted to lead to fixation of both itself and the infection, giving an appearance of a population exhibiting only CI [36].

The Fuchu population of *C. sexmaculata* studied here is polymorphic for the rescue gene. Further investigation of whether this is a balanced or transient polymorphism, and determination of whether the rescue gene imposes a cost on bearers, would provide valuable insight into the dynamics of the spread of suppressors, as well as the generality of the findings in *H. bolina.* Establishment of the frequencies of the rescue gene and male-killer in different populations of *C. sexmaculata* would be valuable. Further, molecular investigation of coccinellids with ecological traits making them liable to male-killer invasion, but in which searches for male-killers using phenotypic assays have proved negative, may reveal presence of beneficial symbionts that are a peaceful resolution of an evolutionary arms race between a male-killer and a suppressor system.

## Methods

### Nomenclature

Lines were designated either Male-killer – Mk, or Normal – N, to reflect the phenotypic status of the P_1_ females in the original sample. The lines were then numbered sequentially within the two categories, hence Mk1 and Mk2 were the two male-killing lines and N1-N13 the 13 normal lines. Subsequent generations show the parental name followed by a ‘.’ to indicate a new generation, and then a number e.g. Mk1.3 is the third cross generated from F_1_ female progeny of Mk1 and Mk1.1.3 is the third cross generated from an F_2_ female, progeny of Mk1.1.

These numbers simply reflect P_1_ phenotype and indicate the matriline. They do not reflect rescue gene status and hence do not necessarily indicate F_1_ (or subsequent generation) phenotype. Rescue gene allelic status is indicated by *res* ^+^ and *res* ^-^ for suppressor and non-suppressor, respectively.

### Production of suppressor free males

For some tests, males known to lack the male-killer suppressor were necessary. These were obtained by tetracycline treatment of singly mated, male-killer bearing females (for both Mk1 and Mk2). Females were mated once and male-killer status confirmed. Females showing characteristic half hatch rates were treated with antibiotic. Where these initial clutches produced only female progeny, and assuming, as was subsequently shown, that *res^+^* was expressed when inherited from the female parent, males produced in the later clutches of these crosses would be homozygous *res^−^*.

### Molecular detection of the male-killer

Different categories of ladybird were assessed for the presence of a bacterial male-killer by performing PCR using general eubacterial primers that amplify the 16S rRNA gene (primer pair 27f, 1495r) [20]. The PCR was carried out using Expand High Fidelity PCR System (Boehringer Mannheim). The product was purified using Microcon Microconcentrators (Amicon Ltd.) and ligated into pGEM T-vector (Promega). The resulting plasmids were transformed into *E. coli* DH5α as described by Hurst *et al.* [21]. Plasmids containing insert DNA were purified using Wizard Minipreps DNA purification system (Promega). Inserts were sequenced using the ABI PRISM BigDye Terminator cycle-sequencing ready-reaction kit (Perkin Elmer) and visualised on an ABI 377 automated sequencer. Primers pUC/M13 forward and reverse, 27f and 1495r and internal primers [37] were used to sequence both strands of the whole unit.

### Phylogenetic analysis

The 16SrDNA sequence generated above was aligned with 16S rDNA sequences from 25 different BLASTN matches with high alignment scores, which were downloaded from the nr database. The accession numbers of the 25 sequences used were: AY296733, CP001277, AF293622, EU348313, AY264676, AF293626, AF293616, AY692361, AY264675, AY136161, AY136136, AY136164, AY136162, AY136163, AY136145, AY136156, AY136148, EU178101, AB273745, AM403659, FM955884, AL590842, CP001048, NR028786, U90757. Sequences were aligned with ClustalW2 [38], minor manual adjustments were made using Seaview [39], and a neighbour-joining tree was generated excluding sites with a gap in any sequence, using Kimura's 2 parameter correction, with 1000 bootstrapped replicates, implemented in ClustalW2 [38]. The tree was displayed using NJPlot [40].

### Calculation of expected progeny ratios

Under a model where the suppressor is a single gene, with alleles *res* ^+^ and *res* ^−^, where *res* ^+^ is a dominant rescue allele and where the vertical transmission efficiency of the male-killer is between 0.8–1.0, the expected progeny ratios from different crosses can be calculated as follows:

In all cases female progeny will survive, whether infected or not; what varies is the proportion of males that inherit the infection and further the proportion of those inheriting the infection which carry a *res* ^+^ allele and so (under this model) survive. If the proportion of progeny inheriting the male-killer varies between 0.8 and 1.0 a maximum of 20% of the progeny (10% male and 10% female) will lack the male-killer.

In a cross where both parents are free from the suppressor (*res* ^−^ *res* ^−^ x *res* ^−^ *res* ^−^) and the parental female is infected with the male-killer, none of the progeny will inherit a suppressor and hence all the male progeny that receive the male-killer will die. This will be between 80 and 100% of the males, i.e. if vertical transmission is 1, 0 males will survive, if the vertical transmission is 0.8, 20% of the progeny will not inherit the male-killer, 10% of these are male, and will now survive, increasing the sex ratio to 0.1/0.6 = 0.167.

Similarly if the parents are *res* ^−^ *res* ^+^ x *res* ^−^ *res* ^−^ half the progeny will inherit a suppressor allele. If vertical transmission is 100% one quarter of the progeny will die (males with no suppressor), and one third, 0.333, of the remaining progeny will be male. If vertical transmission is 80% this would mean of the 20% of the progeny that fail to inherit the male-killer, 10% will be male (as above), of which half will be *res* ^−^ and so will now survive, increasing the proportion male to 0.3/0.8 =  0.375.

If the parents are *res* ^+^ *res* ^−^ x *res* ^+^ *res* ^−^ three-quarters of the progeny will inherit a suppressor so 3/7 or 0.429 of the surviving progeny will be male assuming all inherit the male-killer. If vertical transmission is 80%, again 10% of the progeny that fail to inherit the male killer will be male. Now only a quarter of males are *res* ^−^, hence another 2.5% will survive, making the proportion male 0.4/0.9 = 0.444.

Finally, if one parent is homozygous *res* ^+^ then all progeny inherit a copy of the suppressor, all will survive and the proportion male will be 0.5, regardless of the vertical transmission efficiency.

These calculations assume that in the absence of the male-killer the sex ratio would be 1∶1, that the male-killer is inherited equally by males and females and that it always kills males unless there is a suppressor present.

### Simulations

Simulations were carried out to estimate the 95% confidence limits of the expected sex ratios. This can be illustrated using Mk 1.7. Here the range of possible sex ratios is from 0–0.167, and the sample size is 12. First, a random number is chosen, and on the basis of this, a sex ratio is randomly chosen from an even distribution from 0 to 0.167. Then, using this sex ratio, a binomial sample of 12 individuals is created, of which between 0 and (theoretically) 12 will be male. This process was repeated 100,000 times, to produce a distribution of the numbers of males seen in samples of 12. In this case the distribution is:

0 males: 41.8%

1 male: 30.7%

2 males: 17.2%

3 males: 7.3%

4 males: 2.3%

5 males: 0.5%

6 males: 0.1%

From these values it is concluded that any number of males in the data above 3 would give significant evidence against the hypothesis, since this has a chance of happening that is below 5%, and thus the 95% confidence limits run from zero to three males from 12, or from 0–0.25 as the sex ratio. Corresponding calculations were carried out for each cross listed in Table 2.

### Accession number mentioned in text

EMBL: AJ272038

## Supporting Information

Table S1 Data upon which Figure 2 is based.
(0.04 MB DOC)

Table S2 Details of parents for matrilines of *C. sexmaculata*.
(0.04 MB DOC)
