# Supplementary material for: Intergenomic Arms Races: Detection of a Nuclear Rescue Gene of Male-Killing in a Ladybird
Source: PLoS Pathog. 2010 Jul 8;6(7):e1000987. doi: 10.1371/journal.ppat.1000987 (PMC2900309; doi:10.1371/journal.ppat.1000987)
Supplement: Table S1 — Data upon which Figure 2 is based. (0.04 MB DOC) [file ppat.1000987.s001.doc]

Table S1. Data upon which Figure 2 is based. Effect of second male on sex ratio in Mk1 F_1_ crosses. Progenic sex ratios given for sequential four day periods.

| Female | Male and days | Number of progeny | Proportion male |
| --- | --- | --- | --- |
| Mk1.1 | Male 1 | 75 | 0.013 |
|  | Male 2, days 1-4 | 22 | 0.227 |
|  | Male 2, days 5-8 | 25 | 0.28 |
|  | Male 2, days 9-12 | 27 | 0.444 |
|  | Male 2, days 13-16 | 11 | 0.636 |
|  | Male 2, days 17-20 | 22 | 0.409 |
|  | Male 2, Total | 107 | 0.355 |
| Mk1.2 | Male 1 | 69 | 0.464 |
|  | Male 2, days 1-4 | 28 | 0.5 |
|  | Male 2, days 5-8 | 26 | 0.269 |
|  | Male 2, days 9-12 | 21 | 0.190 |
|  | Male 2, days 13-16 | 18 | 0.111 |
|  | Male 2, days 17-20 | 9 | 0.222 |
|  | Male 2, Total | 102 | 0.284 |
| Mk1.5 | Male 1 | 83 | 0.470 |
|  | Male 2, days 1-4 | 17 | 0.412 |
|  | Male 2, days 5-8 | 56 | 0.289 |
|  | Male 2, days 9-12 | 21 | 0.476 |
|  | Male 2, days 13-16 | 14 | 0.357 |
|  | Male 2, days 17-20 | 13 | 0.353 |
|  | Male 2. Total | 121 | 0.347 |
