# Supplementary material for: Intergenomic Arms Races: Detection of a Nuclear Rescue Gene of Male-Killing in a Ladybird
Source: PLoS Pathog. 2010 Jul 8;6(7):e1000987. doi: 10.1371/journal.ppat.1000987 (PMC2900309; doi:10.1371/journal.ppat.1000987)
Supplement: Table S2 — Details of parents for matrilines of C. sexmaculata. (0.04 MB DOC) [file ppat.1000987.s002.doc]

Table S2. Details of parents for matrilines of *C. sexmaculata* listed in Table 2

| Cross | Female parent | Male parent |
| --- | --- | --- |
| Mk1.1 | Mk1 F_1_ (*res-res-* ) | N5 F_1_ (*res-res-* ) |
| Mk1.1* | Mk1 F_1_ (*res-res-* ) | N1 F_1_ (*res^+^res^+^* ) |
| Mk2.3 | Mk2 F_1_ (*res-res-* ) | N5 F_1_ (*res^+^res-* ) |
| Mk 1.7 | Mk1 F_1_ (*res-res-* ) | N5 F_1_ (*res-res-* ) |
| Mk1.8 | Mk1 F_1_ (*res-res-* ) | N8 F_1_ (*res-res-* ) |
| Mk1.11 | Mk1 F_1_ (*res-res-* ) | N11 F_1_ (*res-res-* ) |
| Mk1.1.1 | Mk1.1 F_1_ (*res-res-* ) | N5 F_1_ (*res-res-* ) |
| Mk 1.1.2 | Mk1.1 F_1_ (*res-res-* ) | N5 F_1_ (*res-res-* ) |
| Mk1.1.3 | Mk1.1 F_1_ (*res-res-* ) | N5 F_1_ (*res-res-* ) |
| Mk1.1.4 | Mk1.1 F_1_ (*res-res-* ) | N5 F_1_ (*res^+^res-* ) |
| Mk1.1*.5 | Mk1.1 F_1_ (*res^+^res-* ) | N5 F_1_ (*res-res-* ) |
| Mk1.10 | Mk1 F_1_ (*res-res-* ) | N4 F_1_ (*res^+^res-* ) |
| Mk1.1*.6 | Mk1.1 F_1_ (*res^+^res-* ) | N5 F_1_ (*res^+^res-* ) |
| Mk1.1.4.9 | Mk1.1.4 F_1_ (*res^+^res-* ) | Mk1.1*.5 F_1_ (*res^+^res-* ) |
| Mk2.3.3.1 | Mk2.3 F_2_ (*res^+^res^+^*) | Mk1 F_2_ (*res-res-* ) post-tet |
| Mk2.3.1.2 | Mk2.3 F_2_ (*res-res-* ) | N1 F_1_ (*res^+^res^+^*) |
